# Prevalence and Incidence of HIV in a Rural Community-Based HIV Vaccine Preparedness Cohort in Masaka, Uganda

**DOI:** 10.1371/journal.pone.0020684

**Published:** 2011-06-03

**Authors:** Eugene Ruzagira, Symon Wandiembe, Andrew Abaasa, Jonathan Levin, Agnes Bwanika, Ubaldo Bahemuka, Matthew A. Price, Anatoli Kamali

**Affiliations:** 1 Medical Research Council (MRC), Uganda Virus Research Institute (UVRI), Uganda Research Unit on AIDS, Entebbe, Uganda; 2 International AIDS Vaccine Initiative (IAVI), New York, New York, United States of America; University of Rochester, United States of America

## Abstract

**Background:**

Local HIV epidemiology data are critical in determining the suitability of a population for HIV vaccine efficacy trials. The objective of this study was to estimate the prevalence and incidence of, and determine risk factors for HIV transmission in a rural community-based HIV vaccine preparedness cohort in Masaka, Uganda.

**Methods:**

Between February and July 2004, we conducted a house-to-house HIV sero-prevalence survey among consenting individuals aged 18–60 years. Participants were interviewed, counseled and asked to provide blood for HIV testing. We then enrolled the HIV uninfected participants in a 2-year HIV sero-incidence study. Medical evaluations, HIV counseling and testing, and sample collection for laboratory analysis were done quarterly. Sexual risk behaviour data was collected every 6 months.

**Results:**

The HIV point prevalence was 11.2%, and was higher among women than men (12.9% vs. 8.6%, P = 0.007). Risk factors associated with prevalent HIV infection for men were age <25 years (aOR = 0.05, 95% CI 0.01–0.35) and reported genital ulcer disease in the past year (aOR = 2.17, 95% CI 1.23–3.83). Among women, being unmarried (aOR = 2.59, 95% CI 1.75–3.83) and reported genital ulcer disease in the past year (aOR = 2.40, 95% CI 1.64–3.51) were associated with prevalent HIV infection. Twenty-one seroconversions were recorded over 2025.8 person-years, an annual HIV incidence of 1.04% (95% CI: 0.68–1.59). The only significant risk factor for incident HIV infection was being unmarried (aRR = 3.44, 95% CI 1.43–8.28). Cohort retention after 2 years was 87%.

**Conclusions:**

We found a high prevalence but low incidence of HIV in this cohort. HIV vaccine efficacy trials in this population may not be feasible due to the large sample sizes that would be required. HIV vaccine preparatory efforts in this setting should include identification of higher risk populations.

## Introduction

The best long-term hope to control the HIV/AIDS pandemic is a safe, effective, and affordable preventive vaccine [1]. HIV vaccine efficacy clinical trials must be performed in African populations where the burden of HIV remains greatest [2] and the need for an effective vaccine most pressing. However, before such trials are initiated, preparatory studies that include estimates of HIV prevalence and incidence need to be conducted in potential study populations [3]. HIV incidence data are necessary to ensure that planned HIV vaccine efficacy trials will be adequately powered to detect a difference between immunized and placebo recipients. The number of volunteers participating in a phase III trial depends mainly on the incidence of HIV infections in the study population with fewer volunteers required where HIV incidence is high [1].

Since 2003, the MRC/UVRI Uganda Research Unit on AIDS, in collaboration with the International AIDS Vaccine Initiative (IAVI), has been conducting Vaccine Preparatory Studies (VPS) to determine the suitability of potential populations for future HIV vaccine efficacy trials should suitable candidate vaccines become available. The objective of the current study was to determine the HIV prevalence, incidence, and risk factors for HIV infection in a rural community-based HIV vaccine preparedness cohort in Uganda.

## Methods

### Study population

The study was conducted in three neighboring rural communities in Masaka district, Uganda. The communities were selected to participate based on the following criteria; experience of HIV prevention research, previously high HIV prevalence [4] and, presence of a health facility from which some study activities could be conducted. We assumed the HIV prevalence to be between 10% and 20% based on historical data [4], and estimated that a maximum sample size of 1,600 would be required to provide an HIV prevalence estimate with 95% confidence interval width of no more than 2 percentage points.

### Procedures

#### HIV sero-prevalence study

The HIV sero-prevalence survey was performed between February and July 2004. First, a census of all residents in the three communities was conducted. Using the census lists, individuals were then contacted in person and invited to participate in the house-to-house survey study. The survey was conducted one community at a time, until a sufficient sample size had been achieved. Individuals were enrolled if they were aged 18–60 years, clinically healthy and willing to give written informed consent, be tested for pregnancy (females), undergo sexual behavior risk assessment, be counseled and tested for HIV and receive test results.

At enrolment, study information was given, written informed consent obtained and an interviewer administered questionnaire used to collect information on demographics, vaccine knowledge, sexual risk behaviors, medical history and 5 ml of blood drawn for HIV serology. Female participants were asked to provide a urine sample for pregnancy testing. Individuals who were not found at home during the first visit were revisited at least twice to give them a chance to participate. One follow up visit was conducted 1–4 weeks after enrolment to provide HIV test results, conduct post-test and risk reduction counseling and referrals for support and care as necessary.

#### HIV sero-incidence study

The HIV sero-incidence study was conducted between October 2004 and April 2007. Individuals who were found to be HIV uninfected during the sero-prevalence study were invited for screening and enrolment at one of three government health centres closest to their home. Enrollment was offered to healthy HIV negative males and females aged 18–60 years, who were able and willing to give written informed consent, be followed quarterly for 2 years, complete interviewer administered questionnaires on HIV risk factors, HIV comprehension, willingness to participate in HIV vaccine trials, undergo repeated HIV voluntary counseling and testing (VCT) and receive the results, and for females, pregnancy testing.

At enrolment, study information was given, written informed consent obtained and information on demographics and sexual risk behaviors collected using an interviewer administered questionnaire. A medical history was taken, a physical examination performed and VCT done. 15 ml of blood was drawn for HIV and syphilis serology and storage of plasma and serum.

At each quarterly follow up visit, a medical history was taken, a symptom directed physical examination performed and HIV VCT conducted. Collection of specimens and laboratory tests were conducted as for the enrolment visit.

Sexual risk behavior data were collected every 6 months and included data on reported symptoms of reproductive tract infections (RTIs), number of sexual partners, casual sex partners, condom use, alcohol consumption and HIV risk perception.

Interim visits were conducted to perform HIV VCT for presumed exposure to HIV, obtain laboratory test results from previous visits, assess and treat illness, and in response to volunteer requests or tracing efforts.

### Ethics statement

The VPS study protocols and informed consent documents were reviewed and approved by the Uganda Virus Research Institute Science and Ethics Committee and the Uganda National Council of Science and Technology. Prior to enrolment, a detailed discussion of the study information was conducted with each participant, and written informed consent obtained. Participants were provided with outpatient free medical care for common illnesses, HIV risk reduction counseling and condoms at all study visits. HIV infected participants were provided with free CD4/CD8 T-cell counts and those eligible referred for initiation of ART available at no cost. Referrals were also made as necessary for prevention of mother-to-child HIV transmission (PMTCT) and other HIV related care services. Seroconverters in the HIV incidence study were in addition given information about and offered participation in an ongoing acute HIV infection study.

### Laboratory methods

Determine (Abbot Laboratories, Japan) rapid test was used to screen for HIV antibodies. HIV positive results were confirmed by performing two parallel ELISAs (Vironostika Uni-Form II Ag/Ab, BioMerieux, Netherlands & Murex HIV.2.O, Murex Biotech Ltd., UK). Western blot testing (Genetic systems, Biorad Laboratories) was used to resolve discrepant ELISA results. HIV testing was repeated after 2–4 weeks on a fresh specimen if the Western blot test was indeterminate. Syphilis testing was done using the rapid plasma reagin (RPR) test (Biotec). RPR reactive specimens were further tested with the Treponema pallidum Haemaglutination (TPHA) assay (Biotec). Participants were considered to have serological syphilis infection if they tested positive for TPHA and RPR with RPR titres of 1∶4 or greater. ßhCG reagent strips (Bayer Multistix 10SG) were used for urine pregnancy testing.

### Statistical analysis

Data were recorded in MS Access and analysed in Stata 10 (StataCorp, College Station, Texas, USA). Participant characteristics were summarized using frequencies and percentages for categorical variables and means and standard deviations for continuous variables. Chi square and t tests were used to compare demographic characteristics between men and women. Odds ratios and their 95% Confidence Intervals (CI) were used to assess the association between variables and HIV prevalence. Unadjusted (univariable) analyses were conducted and a likelihood ratio test (LRT) used to screen for variables to be included in the adjusted (multivariable) model. Variables that reached a significance level of P<0.1 were selected for inclusion in the multivariable model based on the theory of conceptual frameworks [5]. Sex and age were included and retained in the multivariable model as a priori confounders. Variables were removed from the multivariable model using a backward elimination type algorithm, retaining those that remained significant at P<0.05. Predictors of HIV incidence were determined by fitting unadjusted and adjusted models using a similar algorithm to that used for the HIV prevalence analysis.

## Results

### HIV prevalence study

#### Participant characteristics

A total of 9,078 individuals, 3,261 (36%) of whom were in the 18–60 year age category, was listed in the census. Of those aged 18–60 years, 1,977 (60.6%) were contacted in person and invited to participate in the HIV sero-prevalence survey, 585 (17.9%) were not at home, 698 (21.4%) were not contacted because a sufficient sample size had been achieved and one had died. Of those that were contacted, 1,663 (84.1%) consented to participate, 299 (15.1%) refused, and 15 (0.8%) were excluded due to sickness. Among those that consented to participate; 1,013 (61%) were female, 61% were aged less than 35 years, 69% were currently married, 80% were subsistence farmers and only 13% had attained secondary school or higher education. Compared to men, women were younger (p = 0.051); had no formal education (p<0.001) and were more likely to be subsistence farmers (p<0.001) (Table 1).

**Table 1:** Baseline demographic characteristics of 1663 HIV prevalence study participants in Masaka, Uganda.

	All participants		Men		Women			
	n	%	n	%	n	%	Chi-value	P-value
**Total**	1663	100	650	100	1013	100		
**Mean age (SD)**	33.3 (11.3)		34 (11.2)		32.9 (11.3)		t-test	0.051
**Age group**								
18–24	441	26.5	151	23.2	290	28.6	5.94	0.051
25–34	566	34	230	35.4	336	33.2		
≥35	656	39.5	269	41.4	387	38.2		
**Current marital status**								
Married	1,150	69.2	447	68.8	703	69.4	0.07	0.787
Unmarried¶	513	30.8	203	31.2	310	30.6		
**Education level**								
None	256	15.4	63	9.7	193	19.1	33.34	<0.001
Primary school	1,187	71.4	478	73.5	709	70		
≥Secondary school	220	13.2	109	16.8	111	11		
**Occupation**								
Subsistence farmer	1,327	79.8	430	66.2	897	88.6	123.17	<0.001
Other	336	20.2	220	33.9	116	11.5		

¶Unmarried: separated, widowed and never married.

#### HIV prevalence

The overall HIV point prevalence was 11.2% (95% CI 9.8–12.9). HIV prevalence was significantly higher among women than men (12.9% vs. 8.6%, P = 0.007), and increased with age peaking early among women (25–34 years) and later among men (≥35 years) (table 2). Unmarried women were more likely to be HIV infected than those that were currently married (P<0.001). Also, women who reported two or more sexual partners in the past year were more likely to be HIV infected than those who reported only one sexual partner (P<0.05). Men and women who reported a history of genital ulcer disease in the past year had higher HIV prevalence than those who did not.

**Table 2:** Gender stratified HIV prevalence among 1663 study participants in Masaka, Uganda.

	Men		Women	
	n	Prevalence(%)	n	Prevalence (%)
**Total**	650	8.6	1,013	12.9
**Age group**				
18–24	151	0.7	290	10.3
25–34	230	9.6	336	14.9
≥35	269	12.3	387	13.2
**Current marital status**				
Married	447	8.3	703	9.5
Unmarried¶	203	9.4	310	20.7**
**Level of education**				
None	63	14.3	193	9.3
Primary school	478	8.2	709	13.8
≥Secondary school	109	7.3	111	14.4
**Occupation**				
Subsistence farmer	430	9.1	897	13.4
Other	220	7.7	116	9.5
**Sexual partners in the past year**				
0–1	585	8.2	849	11.5
≥2	65	12.3	164	20.1*
**Condom use in past year**				
No	466	8.8	910	12.3
Yes	184	8.2	103	18.5
**Genital ulcer disease in past year**				
No	473	6.8	707	9.8
Yes	177	13.6*	306	20.3**
**Genital discharge in past year**				
No	574	7.8	690	11.6
Yes	76	14.5	323	15.8

¶Unmarried: separated, widowed and never married;

*P<0.05,

**P<0.001 by Chi square or Fisher's exact test.

Age, marital status and a history of genital ulcer disease were independently associated with prevalent HIV infection (table 3). Among men, risk of HIV infection was significantly decreased in the youngest age group i.e. <25 years (aOR = 0.05, 95% CI 0.01–0.35) and increased in those reporting genital ulcer disease in the past year (aOR = 2.17, 95% CI 1.23–3.83). Among women, being unmarried (aOR = 2.59, 95% CI 1.75–3.83) and reported genital ulcer disease in the past year (aOR = 2.40, 95% CI 1.64–3.51) were associated with an increased risk of HIV infection.

**Table 3:** Logistic regression analysis of gender-specific risk factors for HIV infection among 1663 participants in Masaka, Uganda.

	Men				Women			
	OR	95% CI	aOR	95% CI	OR	95% CI	aOR	95% CI
**Age**	1.03*	1.01–1.05	-	-	1.01	0.99–1.02	-	-
**Age group**								
18–24	0.048*	0.01–0.35	0.05**	0.01–0.35	0.76	0.47–1.23	0.95	0.58–1.57
25–34	0.76	0.43–1.34	0.75	0.42–1.33	1.15	0.75–1.75	1.35	0.87–2.10
≥35	1		1		1		1	
**Current marital status**								
Married	1				1		1	
Unmarried¶	1.14	0.64–2.04	-	-	2.47*	1.7–3.59	2.59**	1.75–3.83
**Level of education**								
None	1				1			
Primary	0.53*	0.24–1.16	-	-	1.54*	0.92–2.62	-	-
≥Secondary	0.48	0.17–1.3	-	-	1.64	0.8–3.36	-	-
**Number of sexual partners in the past year**								
0–1	1				1			
≥2	1.57	0.71–3.48	-	-	1.93*	1.25–2.99	-	-
**Condom use in the past year**								
No	1				1			
Yes	0.92	0.5–1.71	-	-	1.61*	0.94–2.75	-	-
**Reported genital ulcer disease in past year**								
No	1		1		1		1	
Yes	2.16*	1.23–3.79	2.17**	1.23–3.83	2.35*	1.62–3.41	2.40**	1.64–3.51
**Reported genital discharge in the past year**								
No	1				1			
Yes	1.99*	0.98–4.04	-	-	1.43*	0.98–2.09	-	-

¶Unmarried: separated, widowed and never married; OR: odds ratio; aOR: adjusted odds ratio;

*P<0.1;

**P<0.05.

### HIV incidence study

#### Participant characteristics

A total of 1,248 individuals were screened and of these 1,184 (95%) enrolled into the HIV incidence study. The most common reasons for study ineligibility were, age above 60 years (26) and failure to comprehend study information (22). Of those enrolled, 722 (61%) were female, 57% were younger than 35 years, 71% were currently married and only 14% had attained secondary school or higher education.

A total of 1,029 (87%) participants completed the study. Of the 155 drop-outs, 121 (78.1%) moved from the study area, 13 (8.4%) lost interest and requested to discontinue participation, 11 (7.1%) were withdrawn after they were recruited into another VPS involving HIV discordant couples, 4 (2.6%) could not be contacted, 3 (1.9%) were terminated due to ill health and 3 (1.9%) died.

Compared with those who completed study follow up, drop-outs were younger (mean age, 32 vs. 35 years; p = 0.006) with most (36%) in the 25–34 year age category, and more likely to have reported condom use in the past year (18.8% vs. 11.3%; p = 0.001) but were otherwise similar in regard to other baseline characteristics.

#### HIV incidence

Twenty one participants seroconverted during 2025.8 person years of follow up, an overall HIV incidence of 1.04% (95% CI: 0.68–1.59). Women had a lower annual HIV incidence (0.8%) compared to men (1.41%) although this difference was not statistically significant (aRR = 0.51, 95% CI 0.21–1.21) (Table 4). The only significant risk factor for HIV acquisition was being unmarried (aRR = 3.44, 95% CI 1.43–8.28).

**Table 4:** Poisson regression analysis of baseline risk factors for incident HIV infection among 1184 participants in Masaka, Uganda.

	n (%)	Seroconverters/PYO	Rate/100 PYO	RR (95% CI)	LRT P-value	aRR (95% CI)
**Total**	1184 (100)	21/2025.8	1.04			
**Sex**						
Male	462 (39)	11/779.9	1.41	1	P = 0.198	1
Female	722 (61)	10/1245.9	0.8	0.57 (0.24–1.34)		0.51 (0.21–1.21)
**Age (mean, SD)**	34 (10.9)			0.99 (0.95–1.02)	P = 0.523	
**Age group**						
18–24	241 (20.4)	2/400.4	0.499	1	P = 0.246	1
25–34	434 (36.6)	11/740.6	1.49	2.97 (0.99–13.42)		4.16 (0.90–19.30)
≥35	509 (43)	8/884.8	0.9	1.81 (0.38–8.53)		2.09 (0.44–9.97)
**Current marital status**						
Married	835 (70.5)	10/1,439.3	0.69	1	P = 0.025	1
Unmarried¶	349 (29.5)	11/586.5	1.88	2.7 (1.15–6.36)		3.44 (1.43–8.28)
**Level of education**						
None	154 (13)	1/265.1	0.38	1	P = 0.441	
Primary	869 (73.4)	17/1495.8	1.14	3.01 (0.4–22.6)		-
≥Secondary	161 (13.6)	3/264.9	1.13	3 (0.31–8.86)		-
**Frequency of alcohol consumption**						
Does not drink	546 (46.1)	8/921.6	0. 87	1	P = 0.294	
Once/month	365 (30.8)	5/639.3	0.78	0.9 (0.29–2.75)		-
≥4 times/month	273 (23.1)	8/464.9	1.72	1.98 (0.74–5.28)		-
**Number of sexual partners in the past year**						
0–1	958 (80.9)	15/1,651.6	0.91	1	P = 0.261	
≥2	226 (19.1)	6/374.2	1.6	1.77 (0.68–4.55)		-
**Concurrent partners**						
No	1,056 (89.2)	16/1,814.5	0.88	1	P = 0.08	
Yes	128 (10.8)	5/211.3	2.37	2.68 (0.98–7.32)		-
**Condom use in past year**						
No	841 (74.2)	13/1460.1	0.89	1	P = 0.176	
Yes	292 (25.8)	8/479.6	1.67	1.87 (0.78–4.52)		-
**Reported genital ulcer disease in past year**						
No	843 (71.2)	13/1450.3	0.9	1	P = 0.339	
Yes	341 (28.8)	8/575.5	1.39	1.55 (0.64–3.74)		-
**Reported genital discharge in past year**						
No	848 (71.6)	16/1449.8	1.1	1	P = 0.633	
Yes	336 (28.4)	5/576	0.87	0.79 (0.29–2.15)		-
**Serological syphilis**						
Positive	23 (1.9)	0/404.8	0	1		
Negative	1,161 (98.1)	21/1985.3	1.06	-	-	-
**Circumcised**						
No	399 (86.4)	10/678.5	1.47	1	P = 0.685	
Yes	63 (13.6)	1/101.4	0.99	0.67 (0.09–5.23)		**-**

¶Unmarried: separated, widowed and never married; PYO: person-years of observation; RR: rate ratio; aRR: adjusted rate ratio.

## Discussion

We found a high HIV prevalence (11.2%) but relatively low incidence (1.04 per 100 person-years) in this rural community-based HIV vaccine preparedness cohort. The observed HIV prevalence was higher than the national average of 6.4% [6]. However, this was expected since we purposely selected communities with previously high HIV prevalence [4] to participate in this study. The HIV incidence in our study was slightly higher than that reported in other rural community-based studies in Uganda [7], [8]. This may be partly because these studies included individuals younger than 18 years in whom HIV incidence rates are relatively low [9], [10].

Consistent with other studies in Sub Saharan Africa [8], [10], [11], [12], HIV prevalence was higher among women than in men. This disparity was more marked in the youngest age group (18–24 years) in which women had a 15-fold higher HIV prevalence compared to men. High rates of HIV infection among young women have been attributed to a higher prevalence of herpes simplex type II infection [13] and early marriage [14]. Conversely, although men had higher HIV incidence than women, this difference was not significant. Available literature on gender-specific HIV transmission risk is inconclusive. Some studies have reported similar HIV transmission risk in both genders [6] while in others, HIV incidence was found to be higher in women compared to men [7], [8]. The reasons for these inconsistent results are not well understood.

Marriage has been reported as risk factor for HIV infection in sub-Saharan Africa [9], [10], [11]. However, in our study, marriage did not increase risk for prevalent HIV infection among men and was even protective among women. Additionally, unmarried participants had an increased risk of HIV acquisition compared to those that were married. This is probably because unmarried participants engaged in riskier behavior compared to those who were married. Indeed, unmarried participants in our study were more likely to report ≥2 sexual partners in the past year than those that were married (data not shown). Also, in a population-based study in the neighboring communities, unmarried men and women were more likely to report a casual partner in the past year than their married counterparts [12].

Genital ulcerative disease (GUD) increases susceptibility to HIV by disrupting the mucosal barrier [13]. In the current study, a history of GUD in the past year was associated with higher HIV prevalence. Although the incidence rate of HIV was higher amongst participants with a history of genital ulcer disease in the past one year, the association was not statistically significant, possibly due to the small number of HIV incident cases.

In addition to providing accurate estimates of HIV incidence, vaccine preparatory studies are necessary to assess recruitment and retention of volunteers over several years and the willingness of a population to participate in future efficacy trials [14]. In the current study, about 87% of participants were retained in follow up for up to two years. Also, willingness to participate in future HIV vaccine efficacy trials in this population is high [15]. However, the low HIV incidence observed in this study implies that vaccine trials in this population would require very large sample sizes. Large community-based trials would require more funding than that which has been available [14]. On the other hand, identification of high-risk groups with higher HIV incidence in rural African communities with generalized epidemics such as ours is difficult [16]. Nevertheless, to ensure participation in future HIV vaccine efficacy trials in these settings, preparedness efforts should include the identification of high risk sub-populations such as individuals in discordant couple relationships and commercial sex workers with higher HIV incidence. The nature of HIV vaccination programs will to some extent be determined by the stage of the HIV epidemic [17]. Whereas countries with concentrated epidemics may target high-risk groups for vaccination, this approach may not be suitable in countries with generalised epidemics [17]. Therefore, although targeting high-risk groups may be practical for evaluating HIV vaccine efficacy in our setting, it may not be appropriate for future HIV vaccination programs.

A major limitation of this study is that sexual risk behaviour data was obtained by self report. We ensured the confidentiality of participants, and that sexual risk behaviour assessments were conducted by well trained and experienced interviewers. However, participants could have under-reported stigmatized behaviours and over-reported socially acceptable behaviours. Another limitation is that routine laboratory RTI screening was limited to syphilis serology in the VPS protocol. Laboratory diagnosis of other RTIs would have allowed for better characterization of the participants' HIV risk profile. Selection bias is also a possible limitation as individuals who did not participate in the study may have had a different HIV risk profile from those who did. In particular, individuals with self-perceived high HIV risk may have refused to or avoided participation in the study, which may have resulted in underestimation of HIV infection rates. Related to this, loss to follow up could have resulted in underestimation of HIV incidence. The most common reason for loss to follow up was out-migration. Migration has been associated with an increased risk of HIV infection probably due to higher risk sexual behavior among those who move [18]. Also, most of the participants who dropped out of the study were in the 25–34 year age group, a category with the highest HIV incidence.

In summary, we found a high HIV prevalence but a low HIV incidence in this rural community-based HIV vaccine preparedness cohort. The low HIV incidence suggests that a vaccine efficacy trial in this population may not be feasible due to the large sample size that would be required. Preparatory efforts for future HIV vaccine efficacy trials in this and similar settings should include the identification of populations with higher HIV incidence.

## References

[1] Esparza J (2001). An HIV vaccine: how and when?. Bulletin of the World Health Organization.

[2] UNAIDS/WHO (2009). AIDS epidemic update.

[3] Vardas E, Buttò S, Glashoff R, Malnati SM, Poli G (2005). Preparing for phase II/III HIV vaccine trials in
Africa.. Microbes and Infection.

[4] Kamali A, Kinsman J, Nalweyiso N, Mitchell K, Kanyesigye E (2002). A community randomized controlled trial to investigate impact of
improved STD management and behavioural interventions on HIV incidence in
rural Masaka, Uganda: trial design, methods and baseline
findings.. Tropical Medicine and International Health.

[5] Victora GC, Huttly RS, Fuchs CS, Olinto AMT (1997). The Role of Conceptual Frameworks in Epidemiological Analysis: A
Hierarchical Approach.. International Journal of Epidemiology.

[6] Quinn TC, Wawer MJ, Sewankambo N, Serwadda D, Li C (2000). Viral Load and Heterosexual Transmission of Human
Immunodeficiency Virus Type 1.. New England Journal of Medicine.

[7] Carpenter LM, Kamali A, Ruberantwari A, Malamba S, Whitworth J (1999). Rates of HIV transmission within marriage in rural Uganda in
relation to the HIV sero-status of the partners.. AIDS.

[8] Hugonnet S, Mosha F, Todd J, Mugeye K, Klokke A (2002). Incidence of HIV infection in stable sexual partnerships: A
retrospective cohort study of 1802 couples in Mwanza Region,
Tanzania.. J Acquir Immune Defic Syndr.

[9] Auvert B, Buvé A, Ferry B, Caraël M, Morison L (2001). Ecological and individual level analysis of risk factors for HIV
infection in four urban populations in sub-Saharan Africa with different
levels of HIV infection.. AIDS.

[10] Sateren WB, Foglia G, Renzullo PO, Eslon L, Wassuna M (2006). Epidemiology of HIV-1 Infection in Agricultural Plantation
Residents in Kericho, Kenya: Preparation for Vaccine Feasibility
Studies.. J Acquir Immune Defic Syndr.

[11] Amornkul NP, Vandenhoudt H, Nasokho P, Odhiambo F, Mwaengo D (2009). HIV Prevalence and Associated Risk Factors among Individuals Aged
13–34 Years in Rural Western Kenya.. Plos One.

[12] Biraro S, Shafer LA, Kleinschmidt I, Wolff B, Karabalinde A (2009). Is Sexual risk taking behaviour changing in rural south-west
Uganda? Behaviour trends in a rural population cohort
1993–2006.. Sexually Transmitted Infections.

[13] Dickerson MC, Johnston JBA, Delea TE, White A, Andrews E (1996). The Causal Role for Genital Ulcer Disease as a Risk Factor for
Transmission of Human Immunodeficiency Virus: An Application of the Bradford
Hill Criteria.. Sexually Transmitted Diseases.

[14] Esparza J, Burke D (2001). Epidemiological considerations in planning HIV preventive vaccine
trials.. AIDS.

[15] Ruzagira E, Wandiembe S, Bufumbo L, Levin J, Price AM (2009). Willingness to participate in preventive HIV vaccine trials in a
community-based cohort in south western Uganda.. Tropical Medicine and International Health.

[16] Kamali A, Quigley M, Nakiyingi J, Kinsman J, Kengeya-Kayondo J (2003). Syndromic management of sexually-transmitted infections and
behaviour change interventions on transmission of HIV-1 in rural Uganda: a
community randomised trial.. The Lancet.

[17] Stover J, Garnett GP, Seitz S, Forsythe S (2002). The Epidemiological Impact of an HIV/AIDS Vaccine in Developing
Countries.

[18] Nunn AJ, Wagner H, Kamali A, Kengeya-Kayondo J, Mulder D (1995). Migration and HIV-1 seroprevalence in a rural Ugandan
population.. AIDS.

